# Population Dynamics, Distribution, and Species Diversity of Fruit Flies on Cucurbits in Kashmir Valley, India

**DOI:** 10.1673/031.013.6501

**Published:** 2013-06-30

**Authors:** S. A. Ganie, Z. H. Khan, R. A. Ahangar, H. A. Bhat, Barkat Hussain

**Affiliations:** Sher-e-Kashmir University of Agricultural Sciences and Technology, Kashmir, Shalimar, Jammu and Kashmir, India

**Keywords:** abiotic factors, abundance, fruit fly population

## Abstract

Given the economic importance of cucurbits and the losses incurred by fruit fly infestation, the population dynamics of fruit flies in cucurbit crops and the influence of abiotic parameters, such as temperature, relative humidity, rainfall, and total sunshine hours per day on the fruit fly population were studied. The study was carried out at six locations; in district Srinagar the locations were Batmaloo, Shalimar, and Dal, while in district Budgam the locations were Chadoora, Narkara, and Bugam (Jammu and Kashmir, India). Various cucurbit crops, such as cucumber, bottle gourd, ridge gourd and bitter gourd, were selected for the study. With regard to locations, mean fruit fly population was highest (6.09, 4.55, 3.87, and 3.60 flies/trap/week) at Batamaloo and Chadoora (4.73, 3.93, 2.73, and 2.73 flies/trap/week) on cucumber, bottle gourd, ridge gourd, and bitter gourd, respectively. The population of fruit flies was significantly correlated with the minimum and maximum temperature. The maximum species diversity of fruit flies was 0.511, recorded in Chadoora. *Bactrocera cucurbitae* (Coquillett) (Diptera: Tephritidae) was the most predominant species in both Srinagar and Budgam, followed by *B. dorsalis* (Hendel) and *B. tau* (Walker), while *B. scutellaris* (Bezzi) was found only in Chadoora. Results of the present investigation may be utilized in developing a sustainable pest management strategy in the agroecological system.

## Introduction

Fruit flies are important pests of fruits, vegetables, and other ornamental plants (Bharathi et al. 2004). Several biotic factors limit the production and productivity of cucurbits, of which the cucurbit fruit fly, *Bactrocera cucurbitae* (Coquillett) (Diptera: Tephritidae), has been the most prominent pest. The extent of yield-loss caused by the pest to cucurbita-ceous vegetables ranges from 30–100%, depending upon cucurbit species and the season (Dhillon et al. 2005). Maggots feed inside the fruits, but at times also feed on flowers and stems. Generally, the females prefer to lay eggs in soft, tender fruit tissues by piercing the tissue with the ovipositor. A watery fluid oozes from the puncture, which becomes slightly concave with seepage of fluid and transforms into a brown resinous deposit. Sometimes pseudo-punctures (punctures without eggs) have also been observed on the skin of fruit. These punctures reduce the market value of the produce. The eggs are laid into unopened flowers, and the larvae successfully develop in the taproots, stems, and leaf stalks (Weems and Heppner 2001).

Two compounds, methyl eugenol (4-allyl-1, 2-dimethoxybenzene) and cue-lure [4-(pacetoxyphenyl)-2-butanone], play significant roles as attractants for male Tephritid fruit flies (Metcalf 1990). These chemicals are plant-related products derived from phenylpropanoid and related compounds. Methyl eugenol occurs widely as a natural product in the plant kingdom, is a highly potent attractant for the males of *B. dorsalis.* Due to its availability and strong attractant properties, it has been used as a trapping agent in capturing native male fruit flies for population estimation and for pest control. Cue-lure has not been isolated directly as a natural product, except as the hydrolysed derivative, and is a potent male attractant to *B. cucurbitae* (Yong 1990). The number of fruit flies captured with cuelure baited traps correlated positively with abiotic factors, i.e., temperature, humidity, and rainfall (Hasyim et al. 2008). Given the economic importance of cucurbits and the losses incurred by fruit fly infestation, it is important to study the population dynamics of fruit flies and the influence of abiotic parameters such as temperature (minimum and maximum), relative humidity (minimum and maximum), rainfall, and total sunshine hours per day on fruit fly population.

## Materials and Methods

An extensive survey was conducted during 2008 and 2009 in two districts, Srinagar and Budgam, of Kashmir valley (Jammu and Kashmir, India) in order to study the occurrence of fruit flies associated with cucurbitaceous crops. In each district, three locations were selected to be surveyed. In district Srinagar, the locations were Batmaloo, Shalimar, and Dal, while in district Budgam the locations were Chadoora, Narkara, and Bugam. The survey was conducted at weekly intervals on different cucurbit crops such as bottle gourd, cucumber, ridge gourd, and bitter gourd. The trap consisted of a transparent mineral water bottle containing 0.1% methyl eugenol (Hi-Media, www.himedialabs.com) with > 96% purity and dichlorovos. The attractant was in the slow-releasing polymeric plug form. The plugs were dispensed in improvised 500 mL mineral water bottle traps with two windows (3×2 cm) made on opposite sides of the bottles 7 cm from the top. The lid of the trap was perforated, and a nylon thread was knotted and passed through to prevent the thread from slipping through. A thin cotton thread was fastened to the nylon thread from the knotted end, and the polymeric lure plug was tied at the opposite end of the cotton thread. The suspended plug on the cotton thread was held inside the trap at 7 cm from the knot. A strip of dichlorovos was placed at the bottom of the trap to kill insects that entered the trap. The experiment was conducted in a farmer's commercial vegetable field at the previously mentioned locations. At each location, four traps were placed over an area of 0.05 ha. Every week, the number of fruit flies per trap was counted and new traps were put in the field. The collected fruit flies were identified by Professor Viraktmath, Department of Entomology, University of Agriculture Sciences, GKVK Campus, Bangalore, by using the taxonomic keys developed by Billah et al. (2009).

**Table 1.**
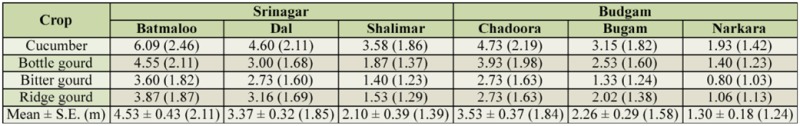
Mean population of fruit flies/trap/week in the Srinagar and Budgam districts. The value represents mean of three replications. Figures in parentheses are square root transformed values. CD (*p* ≤ 0.05), place = 0.325, crops = 0.796, places × crops = 1.562.

Meteorological observations related to maximum temperature, minimum temperature, relative humidity in the morning, relative humidity in the evening, rainfall, and sunshine were taken from the Meteorological Department, Rambagh, and Division of Agronomy, SKUAST-K, Shalimar (Jammu and Kashmir, India), and were correlated with the population of fruit flies.

Species diversity was calculated after completing the identification of collected fruit flies. Species diversity (H) was calculated by the formula given by Margalef (1957) based on Shannon-Wiener function as: H = -Σpi log_10_pi, where pi = Ni/N, Ni = total number of individual in a species, and N = total number of individual in all species Evenness (j) was calculated to estimate the equitability component of diversity using the formula (Pielou 1975): J = H/log_10_S.

Richness (ma) was calculated using the formula: ma = S-1/log_10_, where S = total number of species collected.

### Statistical analysis

Analysis of variance was performed using Statistical Package for Social Sciences (SPSS) software version 16 (SPSS, Inc, www.01.ibm.com/software/analytics/spss), and significant differences among means were reported at 5% level of significance. The data were transformed in order to remove zero counts as a result of no catches in some traps.

## Results

The data (Table 1) revealed that among the various locations selected in district Srinagar, Batmaloo recorded the highest mean population of flies/trap/week, while Shalimar recorded the lowest flies/trap/week in all the test crops. The highest numbers of flies were recorded on cucumber, followed by bottle gourd, ridge gourd, and bitter gourd. Similarly, in district Budgam, Chadoora recorded the highest mean population of flies/trap/week, while Narkara recorded the lowest flies/trap/week in all the cucurbit crops. The highest numbers of flies were observed on cucumber, followed by bottle gourd, ridge gourd, and bitter gourd.

The correlation and multiple regression equation of weather parameters with the population of fruit flies on test crops at selected locations showed that among the various weather parameters studied, minimum temperature was significantly negatively correlated with the population of fruit flies, while maximum temperature, relative humidity in the morning, relative humidity in the evening, rainfall, and sunshine were not significantly correlated with the population of fruit flies (Tables 2, 3). The coefficient of multiple determination (R^2^) de-picted that all the abiotic factors simultaneously affected the mean fruit fly population in Srinagar and Budgam.

**Table 2.**
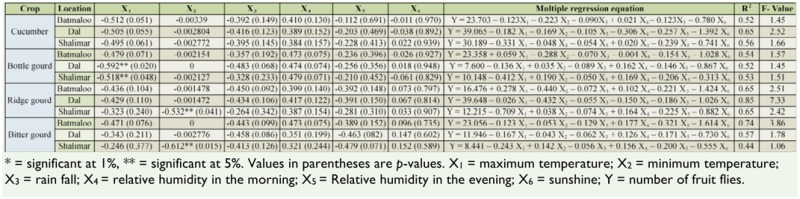
Correlation and multiple regression equation of weather parameters with the population of fruit flies on cucurbits in district Srinagar.

**Table 3.**
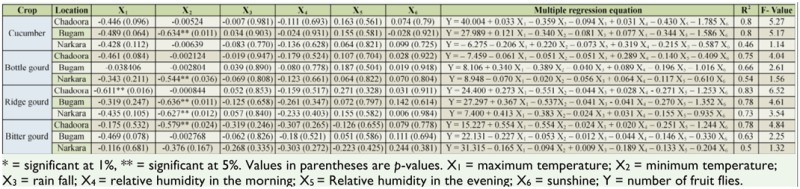
Correlation and multiple regression equation of weather parameters with the population of fruit flies on cucurbits in district Budgam.

**Table 4.**
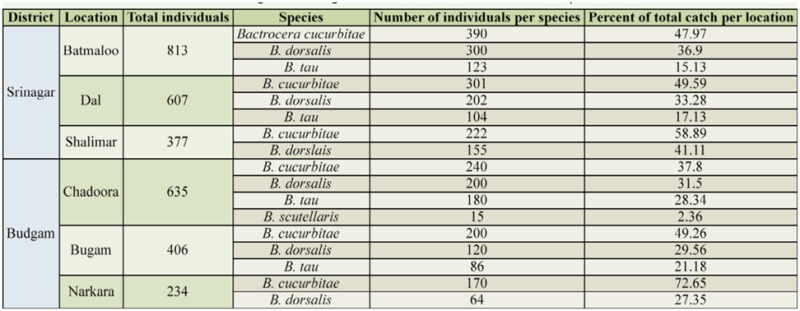
Distribution of fruit flies in the Srinagar and Budgam districts on different cucurbit crops.

Table 4 and 5 present the distribution and bio-diversity of fruit flies in Srinagar and Budgam. The total number of individual flies trapped in Srinagar was highest in Batamaloo, followed by Dal and Shalimar. In Budgam, the number of flies trapped was highest in Chadoora, followed by Bugam and Narkara. The numbers of species trapped in each location are listed in Table 5. In Srinagar, the species diversity was highest in Dal, followed by Batamaloo and Shalimar. In Budgam the species diversity was highest in Chadoora, followed by Bugam and Narkara. Species evenness and richness are listed in Table 5.

**Table 5.**
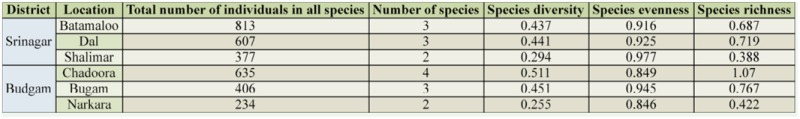
Biodiversity of fruit flies on different cucurbit crops in the Srinagar and Budgam districts.

Table 4 shows the percentage of each species caught in each location. In both Batamaloo and Dal, *B. cucurbitae* made up the highest percentage of captured flies, followed by *B. dorsalis* and *B. tau.* In Shalimar, more *B. cucurbitae* were caught than *B. dorsalis.*

In Chadoora, *B. cucurbitae* made up the highest percentage of captured flies, followed by *B. dorsalis, B. tau,* and *B. scutellaris.* In Bugam, *B. cucurbitae* made up the highest percentage, followed by *B. dorsalis* and *B. tau.* In Narkara, more *B. cucurbitae* were caught than *B. dorsalis.*

Table 5 shows the species diversity and richness in each district. The species diversity was maximum in Chadoora (Budgam), and the species richness in district Srinagar was greatest in Dal and lowest in Shalimar. In district Budgam, the species richness was greatest in Chadoora and lowest in Narkara.

## Discussion

The results of our study indicate that in district Srinagar, Batmaloo recorded the highest mean population of flies/trap/week, while Shalimar recorded the lowest flies/trap/week in all the cucurbit crops. This was mainly because of the fact that the Batmaloo trap area was surrounded by various farms that provided habitat for the fruit flies. In Shalimar, the farms were well-managed and hence did not provide the optimum habitat for fruit flies. These findings are in agreement with that of Vargas et al. (1990). In district Budgam, Chadoora recorded the highest mean population of flies/trap/week, while Narkara recorded the lowest flies/trap/week in all the cucurbit crops. The highest population in Chadoora could be due to the cultivation of local varieties of these crops, while in Bugam and Narkara there is cultivation of hybrid varieties of these crops. These findings are similar to those of Qureshi et al. (2000), who reported that local varieties did not show any satisfactory resistance against fruit flies on cucurbits. The low population in Narkara may also be due to the large cultivation of vegetables and indiscriminate use of pesticides on vegetables, resulting in the reduction of these fruit flies. Between the two districts, Budgam had a lower fruit fly population than Srinagar. This difference could be due to the large-scale cultivation of vegetables in Budgam, and therefore the use of more pesticides and hybrid varieties. Furthermore, Budgam is located at a higher altitude (1610 m a.s.l.) than Srinigar (1585 m a.s.l.).

Among the weather parameters, minimum temperature was significantly negatively correlated with the population of fruit flies, while maximum temperature, relative humidity in the morning, relative humidity in the evening, rainfall, and sunshine were not significantly correlated with the population of fruit flies. The negative correlation of the minimum temperature and population is most likely because the population of fruit flies increases with the ripening of fruits, and cucurbit crops ripen from from July until October, a period in which the minimum temperature decreased in the studied area. These results are similar to that of Liu and Yeh (1982), Shukla and Prasad (1985), and Tariq et al. (2002), who related the population of fruit flies with the ripening of crops. The minimum temperature showing a significant effect and the other abiotic factors reflecting a non-significant affect on the population of fruit flies is in agreement with the results of Raghuvanshi et al. (2012). However, maximum temperature was significantly and negatively correlated with population of fruit flies, which contradicts the results of earlier findings. This difference may be due to variation in agro-ecological system as well as cropping pattern of the experimental location.

Species diversity and species richness were highest in Chadoora and lowest in Narkara, as the highest number of species were found in Chadoora and the lowest were found in Narkara.
